# How I do it: meningioangiomatosis of the sylvian fissure. The need for a tailored functional neurosurgery

**DOI:** 10.1007/s00701-026-07007-x

**Published:** 2026-10-01

**Authors:** Sergio Corvino, Giuseppe Corazzelli, Tamara Ius, Giuseppe Catapano

**Affiliations:** 1 Department of Neurosurgery, “Ospedale del Mare”, 80147 Naples, Italy; 2 Department of Neurosurgery, Santa Maria Delle Grazie”, Pozzuoli, 80078 Naples, Italy; 3 University of Rome La Sapienza, Rome, Italy; 4 Department of Neurosciences, University of Padova, 35121 Padua, Italy; 5 Present Address: Department of Neurosurgery, University of Sassari, 07100 Sassari, Italy

**Keywords:** Meningioangiomatosis, Sylvian Fissure, Functional Neurosurgery, Epilepsy

## Abstract

**Background:**

Meningioangiomatosis is a formidable neurosurgical challenge due to its heterogeneous nature and anatomical localization which often make impossible gross total resection. Surgery is reserved for drug-resistant epileptic seizures aiming their control.

**Method:**

We describe a case of meningioangiomatosis of the right sylvian fissure involving the M1-M2 segments of right middle cerebral artery. A subtotal lesion resection, completed with disconnection surgery through multiple subpial circumferential transections was performed.

**Conclusion:**

To underline the efficacy of the disconnection surgery and the pivotal role of intraoperative electrophysiologic assessment for tailoring the surgical resection at the presumed ictal onset zone.

**Supplementary Information:**

The online version contains supplementary material available at https://doi.org/10.1007/s00701-026-07007-x.

## Relevant surgical anatomy

**Fig. 1:**
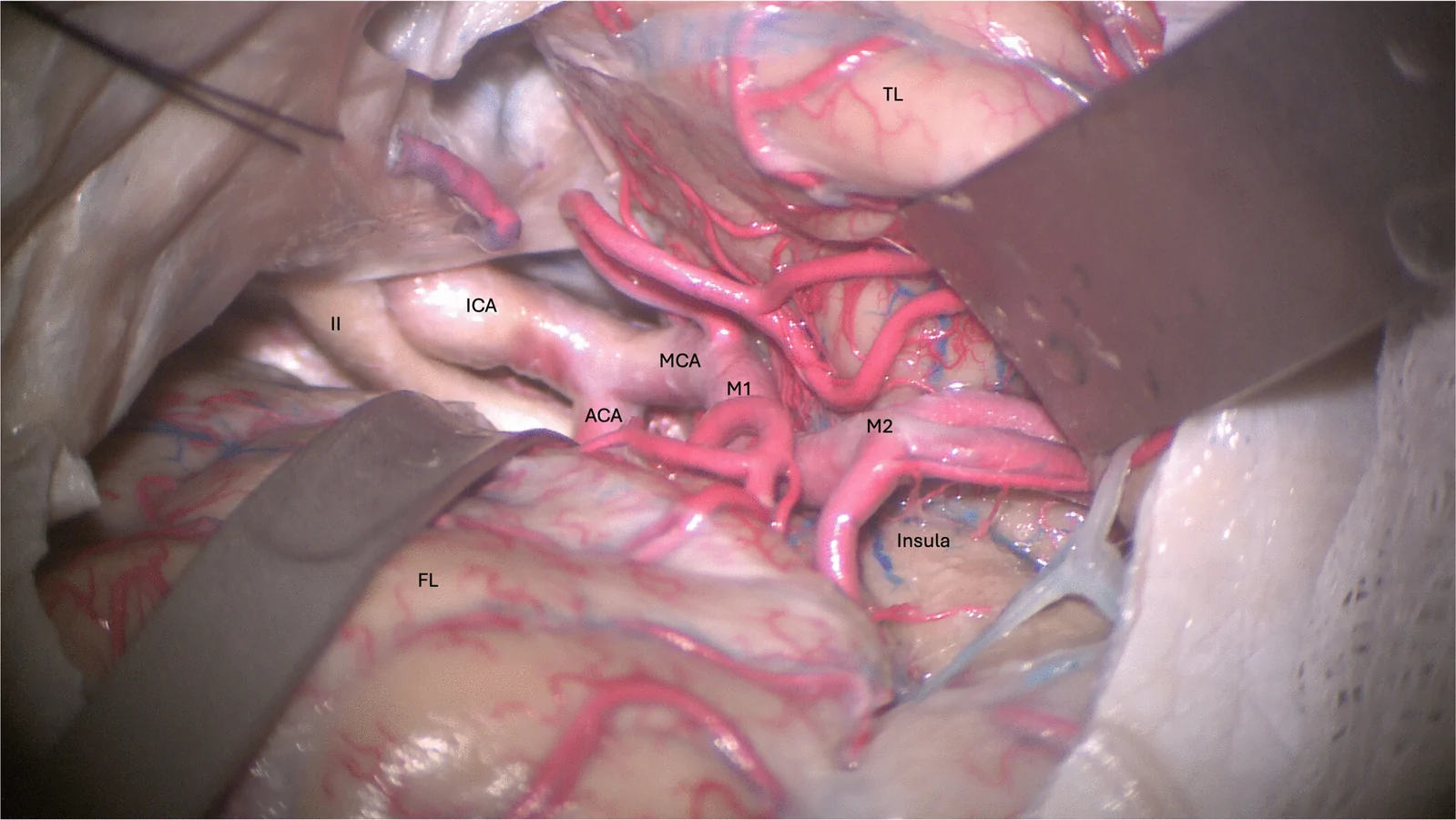
Cadaveric dissection with splitting of right sylvian fissure. TL: Temporal Lobe; FL: Frontal Lobe; ICA: Internal Carotid Artery; ACA: Anterior Cerebral Artery; MCA: Middle Cerebral Artery; M1 and M2: segments of MCA; II: second cranial nerve

Sylvian fissure is a CSF-filled corridor extending from the basal cisterns to the lateral cerebral surface and hosting the middle cerebral artery (MCA) (Fig. 1). It is formed by tightly apposed opercula and is bridged by arachnoid trabeculae and veins, which must be carefully dissected to expose the insula and MCA bifurcation.

We distinguish a superficial and deep part [3]. The superficial part includes a *stem* that begins at anterior clinoid process and extends laterally to the pterion, and *three rami—*anterior horizontal, anterior ascending and posterior – that define the pars orbitalis, pars triangularis and pars opercularis of the inferior frontal gyrus, as well as the limit between the frontal-parietal lobes and temporal lobe. The deep part, also known as sylvian cistern, is divided into *anterior operculoinsular* – or sphenoidal – compartment, that extends laterally from the carotid cistern between the temporal and frontal lobes and carries the M1 segment of MCA, and *lateral operculoinsular* compartment, that includes the area between the opercular and insular clefts, and carries the M2-M3 segments of MCA.

## Description of the technique

In meningioangiomatosis (MA), surgery should primarily target seizure control while preserving neurological function. Microsurgical debulking is performed whenever a safe cleavage plane is present. However, when the lesion is densely adherent to major vessels or eloquent structures, aggressive resection may expose the patient to unacceptable neurological risks. In these situations, a functional disconnection strategy can be adopted. This technique is a function-preserving approach aimed at seizure control rather than complete lesion excision. The strategy focuses on isolating, under continuous neurophysiological monitoring, the epileptogenic cortex by disconnecting the lesion and its involved cortex from surrounding normal brain through circumferential subpial transections and disconnection of afferent and efferent white-matter pathways, while leaving the often diffuse, plaque-like leptomeningeal lesion itself largely in situ, while preserving vascular integrity and eloquent networks. The extent of disconnection is tailored intraoperatively according to anatomical findings and electrophysiological feedback. The operative strategy begins with a wide sylvian fissure dissection aimed at exposing the lesion, defining its relationship with MCA branches and identifying the surrounding epileptogenic cortex. Surgery is performed through a standard pterional craniotomy under continuous intraoperative neurophysiological monitoring. After dural opening, the sylvian fissure is widely split; arachnoid adhesions are sharply dissected and the M1 segment, MCA bifurcation, and M2 branches are progressively exposed. Particular attention is paid to defining the anatomical relationship between the lesion, the MCA branches, and the surrounding operculo-insular cortex. Because of the dense adherence to major arterial branches, aggressive lesion resection is avoided and surgical strategy shifts from lesionectomy to functional disconnection. The presumed epileptogenic cortex surrounding the lesion is identified using preoperative electroclinical data and intraoperative neurophysiological assessment. Circumferential subpial transections are then performed around the lesion, maintaining the dissection immediately beneath the pia mater to preserve cortical vessels and avoid injury to adjacent functional parenchyma. The disconnection proceeds sequentially along the anterior, posterior, superior, and inferior margins of the epileptogenic zone surrounding the lesion. Short U-fibers and cortical association pathways connecting the lesion to the surrounding cortex are interrupted while preserving major vascular structures. Whenever possible, subcortical disconnection is extended to interrupt deeper seizure-propagation pathways identified by anatomical and electrophysiological guidance. Any significant neurophysiological change prompts immediate reassessment of the dissection plane and may limit the extent of resection or disconnection. The procedure is completed once circumferential isolation of the epileptogenic network has been achieved and neurophysiological signals remain stable. Residual lesion portions densely adherent to MCA branches may be intentionally left in situ to avoid vascular injury, provided that functional disconnection has been accomplished.

### Illustrative case

A 22-year-old female was observed for drug-resistant focal epileptic seizures (DRES). Electroencephalography (EEG) showed mild slow bioelectrical anomalies on the right lateral regions.

**Fig. 2:**
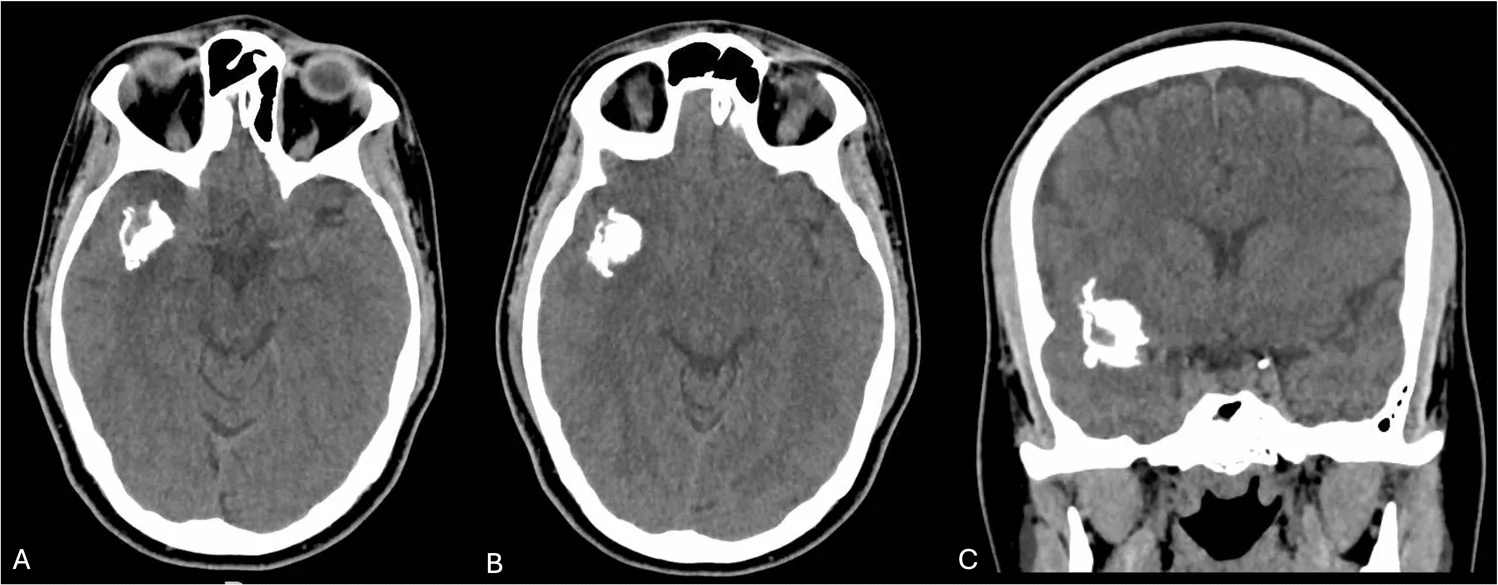
Preoperative computed tomography (CT) of the head. Axial (**A, B**) and Coronal (**C**) sequences. Nodular, cortico-pial calcific mass in the fronto-opercular-insular region of right sylvian fissure surrounded by hypodensity halo

**Fig. 3:**
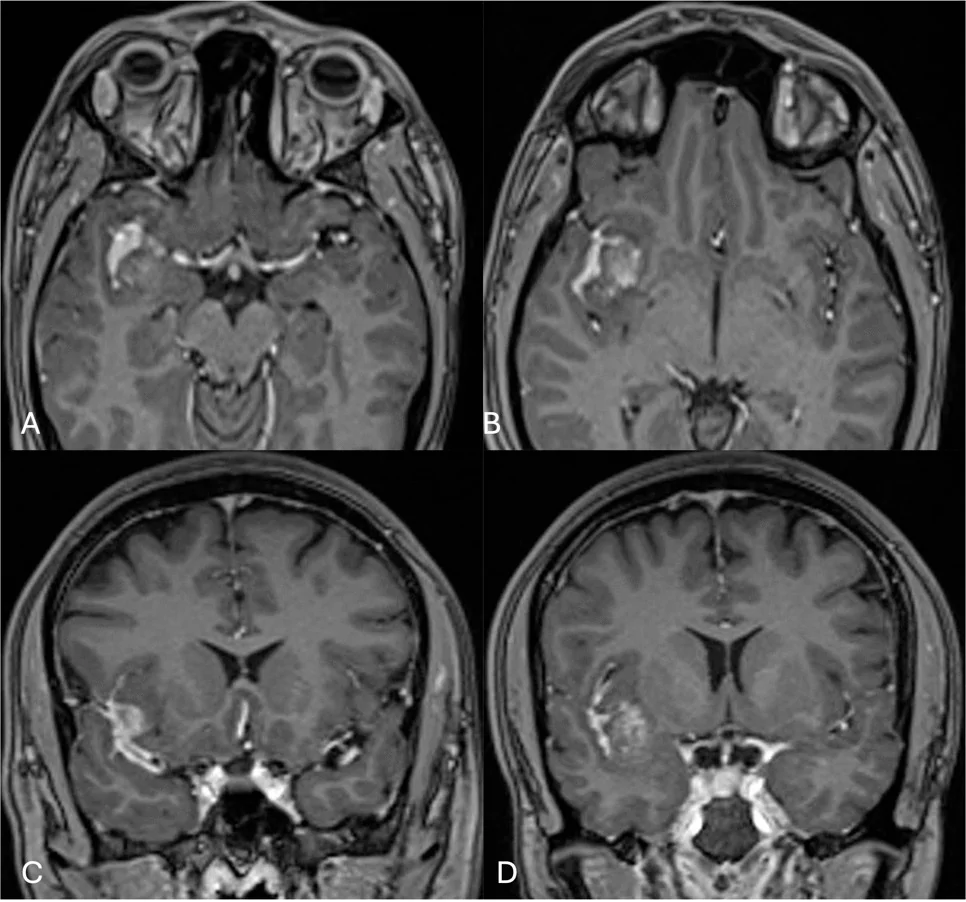
Preoperative contrast-enhanced Magnetic Resonance Imaging (MRI) of the brain. Axial (**A**, **B**) and Coronal (**C**, **D**) sequences. Lesion with slight contrast-enhancement of the overlying cortex and leptomeninges, is firmly attached and covered by two trunks of the M1-M2 segments of right MCA

Computed tomography (CT) (Fig. 2) and contrast-enhanced Magnetic Resonance Imaging (MRI) of the brain (Fig. 3) showed MA of the right sylvian fissure.

**Fig. 4:**
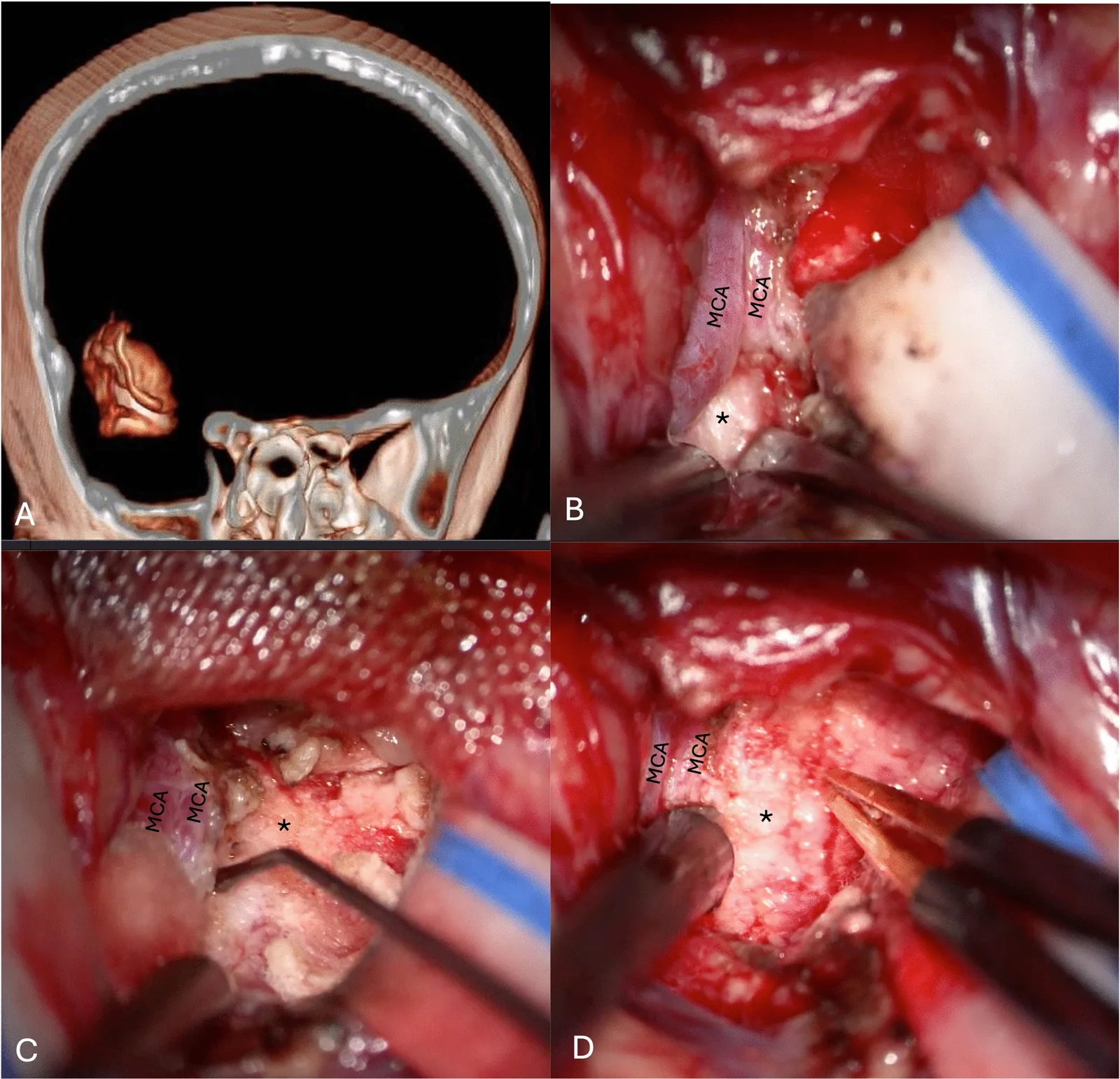
**A**) MRI 3D reconstruction. The two main trunks from M1-M2 segments of right MCA cover the lateral surface of the lesion. **B-D**) Microsurgical intraoperative images. Right side, pterional approach. Gentle separation of the two vascular trunks exposes part of the lesion (*) (B), that is firmly attached to the vessel’s wall (C). After experiencing sudden and profuse bleeding from the lower trunk of M2 segment while attempting to dissect the lesion from the vessel and reduction of motor evoked potential of left hand at IONM, we opted for a disconnection surgery leaving the part of the lesion firmly attached to the vessel’s wall (D). (MCA: Middle Cerebral Artery; *: lesion)

Under intraoperative neurophysiological monitoring (IONM) patient underwent right pterional approach (Video). Once the sylvian fissure was split, a calcific hard nodular mass, firmly attached to and covered by bifurcation trunks of M2 segment of MCA was exposed (Fig. 4). After the initial removal of the parenchymatous component of the lesion, while attempting to dissect the two M2-trunks from the lesion, sudden profuse bleeding from the vessel’ wall occurred, that was promptly stopped with coagulation and surgicel. As transient weakness of the left arm was registered on IONM, we immediately changed surgical strategy, deciding to leave the residual lesion attached to the MCA trunks, but disconnecting it from the brain through multiple subpial circumferential transections with the aim to interrupt the seizures pathway, always under neurophysiological monitoring. At the end of the procedure the neurophysiological signals returned to baseline values.

After surgery patient was without neurological deficits and free from seizures.

At 1 year assessment, she continued to be free from seizures without drug therapy, and the imaging showed the known residual lesion without radiological findings of progression.

## Indications

Meningioangiomatosis is an extremely rare lesion, characterized by leptomeningeal and cortical vascular proliferation [4, 5], representing hard challenge for its extremely heterogeneous nature, behavior and localization [1]. DRES is the most frequent presenting symptom [2] and the neurosurgical indication.

Preoperative management of meningioangiomatosis-related epilepsy is not standardized [2]. Lesionectomy, epileptic surgical resection and biopsy represent the treatment options [2]. Seizures control is achieved in the early postoperative period in 79% and at last follow-up in 88.7% after complete lesion resection and in 64.7% after subtotal resection [2]. Epileptic seizures frequently originate from functionally related perilesional networks rather than from the lesion itself. In this setting, we stress the importance of a tailored functional surgery, where invasive EEG mapping plays a decisive role not only in functional preservation but also in real-time surgical decision-making.

## Limitations

The high density of functional and vital neurovascular structures in and around the sylvian fissure make this surgery particularly demanding. Major limitations include MCA encasement, deep surgical corridor, proximity to eloquent cortex, insular involvement, poor lesion–brain cleavage plane, complex epileptogenic network. All these issues may preclude gross-total resection and require alternative functional strategies.

## How to avoid complications

Safe and successfull MA-surgery requires early identification of the presumed ictal onset zone and its relationships with the intimate neurofunctional areas and neurovascular structures. Preoperative CT scan, contrast-enhanced MRI and EEG are mandatory. Additionally, intraoperative neuronavigation system and continuos electroneurophisiologycal monitoring and mapping guide the procedure.

The adjacent MCA segments, as well as the brain parenchyma, not associated to seizures, must be identified and preserved. When the ictal zone is fully exposed, circumferential dissection begins, and care is taken to stay just superficial in the subpial plane to avoid injury to the adjacent parenchyma.

## Specific information to give to the patient about surgery and potential risks

Patients should be informed about the rationale for treatment. Potential risks of surgical resection include intraoperative hemorrhage from the MCA and postoperative ischemic stroke, as well as injury of eloquent cortex, with consequent severe neurofunctional deficits, and other standard surgical risks.

## Ten key points

1. Meningioangiomatosis is a hamartomatous non-neoplastic lesion.
2. Drug-resistant focal epileptic seizures represent the most frequent presenting symptom and the surgical indication.
3. Surgery aims to achieve seizures control and should be performed as soon as possible once the diagnosis of epilepsy meningioangiomatosis-related is suspected.
4. Failure of safe lesion-vessels dissection drives the conversion from lesionectomy to perilesional epileptogenic disconnection.
5. Surgery must be addressed to the presumed ictal onset zone which is identified by preoperative EEG and confirmed and identified intraoperatively by neuronavigation and electrophysiological monitoring and mapping.
6. Intraoperative electrophysiologic assessment and intra-operative functional brain mapping play a pivotal role, by tailoring the surgical resection of the meningioangiomatosis at the presumed ictal onset zone.
7. Epileptic seizures frequently originate from functionally related perilesional networks rather than from the lesion itself.
8. When gross-total resection is not achievable under safe conditions, a subtotal lesion resection completed by disconnection surgery is a valid alternative option providing immediate and long-term seizure control.

## Supplementary Information

Below is the link to the electronic supplementary material.

Supplementary file1 (MP4 62054 KB)

## Data Availability

No datasets were generated or analysed during the current study.

## References

[1] Giulioni M, Acciarri N, Zucchelli M, Marucci G, Badaloni F, Calbucci F (2006) Meningioangiomatosis involving the wall of the middle cerebral artery. J Neurooncol 78(1):105–106. 10.1007/s11060-005-9066-0 16554969

[2] Roux A, Zanello M, Mancusi RL et al (2021) Meningioangiomatosis: multimodal analysis and insights from a systematic review. Neurology 96(6):274–286. 10.1212/WNL.0000000000011372 33361266

[3] Tanriover N, Rhoton AL, Kawashima M, Ulm AJ, Yasuda A (2004) Microsurgical anatomy of the insula and the sylvian fissure. J Neurosurg 100(5):891–922. 10.3171/jns.2004.100.5.0891 15137609

[4] Tomkinson C, Lu JQ (2018) Meningioangiomatosis: a review of the variable manifestations and complex pathophysiology. J Neurol Sci 392:130–136. 10.1016/j.jns.2018.07.018 30056201

[5] Wiebe S, Munoz DG, Smith S, Lee DH (1999) Meningioangiomatosis. A comprehensive analysis of clinical and laboratory features. Brain 122(Pt 4):709–26. 10.1093/brain/122.4.709 10219783

